# Congenital Bronchobiliary Fistula: A Case Report and Literature Review

**DOI:** 10.3389/fped.2021.686827

**Published:** 2021-08-04

**Authors:** Zhen Bing, Rui Chen, Pengchao Xing, Yueyi Ren, Kefeng Hou

**Affiliations:** Heart Center, Qingdao Women and Children's Hospital, Qingdao, China

**Keywords:** congenital bronchobiliary fistula, bronchoscope, endobronchial blocker, fistulography, thoracoscopic surgery

## Abstract

Congenital bronchobiliary fistula (CBBF) is a rare disease. Children with CBBF mostly have atypical clinical manifestations that can be easily missed. We report a case of a child with CBBF who was diagnosed with fistulography with the help of an endobronchial blocker and a fiberoptic bronchoscope. The CBBF was successfully removed by thoracoscopic surgery.

## Introduction

Congenital bronchobiliary fistula (CBBF) is a rare disease, which was first reported by Neuhauser et al. (1). It is a rare developmental anomaly, i.e., fistula between the respiratory and biliary tract. Its clinical manifestations are recurrent and persistent refractory pulmonary inflammation. We reported a 2-year-old boy with CBBF, who was successfully diagnosed with fistulography and treated by thoracoscopic surgery.

## Case Presentation

**Figure 1:**
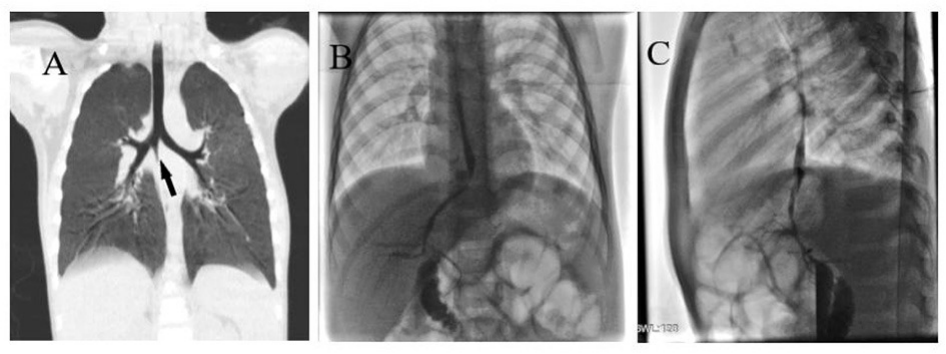
**(A)** Right main bronchus wall with conical cleft-like protrusion (black arrow). **(B)** Anteroposterior angiography of BBF. **(C)** Lateral angiography of BBF.

A 2-year-old boy was hospitalized in our center for recurrent pneumonia. He was hospitalized three times for pneumonia in the past 6 months. The main symptom was cough. The child is too young to cough sputum out. There was no typical yellow-greenish sputum in the period of pneumonia. There was also no cyanosis, respiratory distress, or jaundice. Chest x-ray showed chronic inflammatory changes on the right side at the beginning of the disease. Application of antibiotics and drug-aerosol inhalation can relieve cough symptoms. Chest CT showed multiple patchy hyperdense shadows in both lungs. The 3D-CT reconstruction showed that the wall of the right main bronchus has a conical cleft-like protrusion with the end pointing downward last time (Figure 1A).

**Figure 2:**
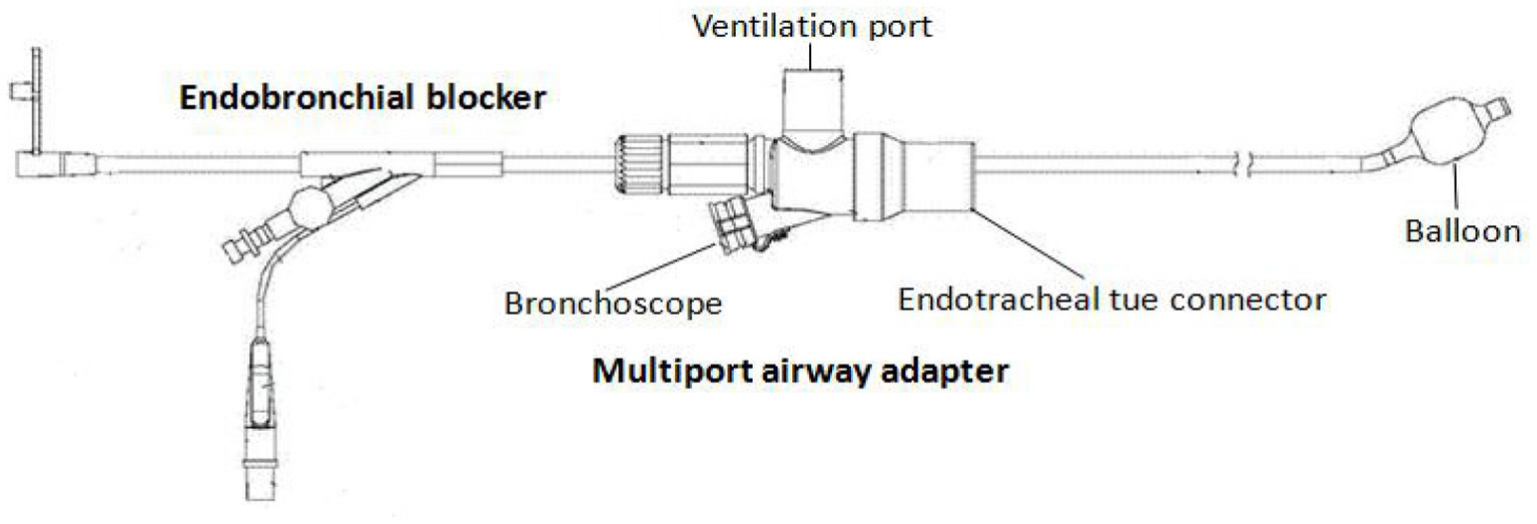
The pediatric endobronchial blocker system.

**Figure 3:**
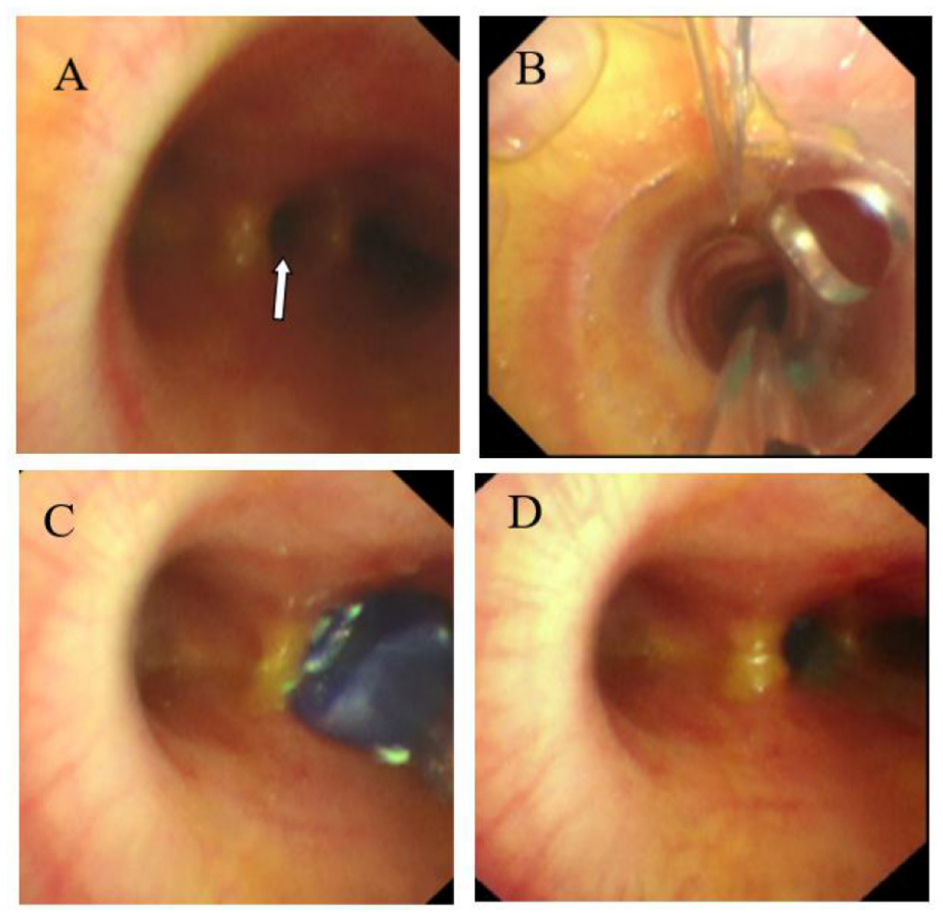
**(A)** Abnormal opening of the tracheal carina (white arrow). **(B)** Endobronchial blocker enter into the trachea. **(C)** Endobronchial blocker enter into the fistula. **(D)** The endobronchial blocker was inflated and fixed after entering the fistula.

In the catheter room, fiberoptic bronchoscopy and bronchography were performed at the same time. Under general anesthesia, a standard 5.5-mm-inner diameter (ID) endotracheal tube (ETT) without cuffed was successfully inserted into the trachea. A multiport airway adapter (Tappa, Common type, EBT0105) was used to connect endotracheal intubation (Figure 2) (2). An electronic fiberoptic bronchoscope was inserted into the trachea through the multiport airway adapter, whose outside diameter is 2.8 mm. We can see yellow secretions attached to the glottis and trachea through the fiberoptic bronchoscope. An abnormal opening was found at the level of the tracheal carina, and yellow secretions could be seen at the opening (Figure 3A). The pediatric endobronchial blocker (5-Fr) was inserted into the tube through the multiport airway adapter. Different models of endobronchial blockers have clear matching endotracheal intubation diameter and fiberoptic bronchoscope specification (Table 1). With the assistance of an electronic fiber bronchoscope, the endobronchial blocker was inserted into the fistula (Figures 3B–D). Two milliliters of gas was injected into the endobronchial blocker through the end lateral hole, and the cuff of the endobronchial blocker was filled to close the fistula. Iodixanol was used as the contrast agent. A 20-ml syringe was used to connect the exhaust pipe of the endobronchial blocker to inject the fistula, and DSA was performed at the same time. It can be seen that the contrast medium is connected to the left hepatic duct through the esophageal hiatus, and the contrast medium sequentially fills the left intrahepatic bile duct, the right intrahepatic bile duct, the common hepatic duct, the common bile duct, the gallbladder, and the duodenum (Figures 1B,C). No other biliary malformation was found. Ultrasound examination of the liver and gallbladder showed no abnormality. The examination of hydatidosis and other parasites was normal. By way of aspiration of intratracheal lavage fluid examination, the total bile acid was found to be 306.20 μmol/L. Esophagotracheal fistula was not found by upper gastrointestinal angiography.

**Table 1:** Different models of endobronchial blocker have clear matching endotracheal intubation diameter and fiberoptic bronchoscope specification.

Endobronchial blocker (Fr)	5			7			9		
Endotracheal intubation (mm)	4.5	5.0	5.5	6.0	6.5	7.0	7.5	8.0	8.5
Bronchoscope (mm)	2.2		2.8	3.5			4.0		

**Figure 4:**
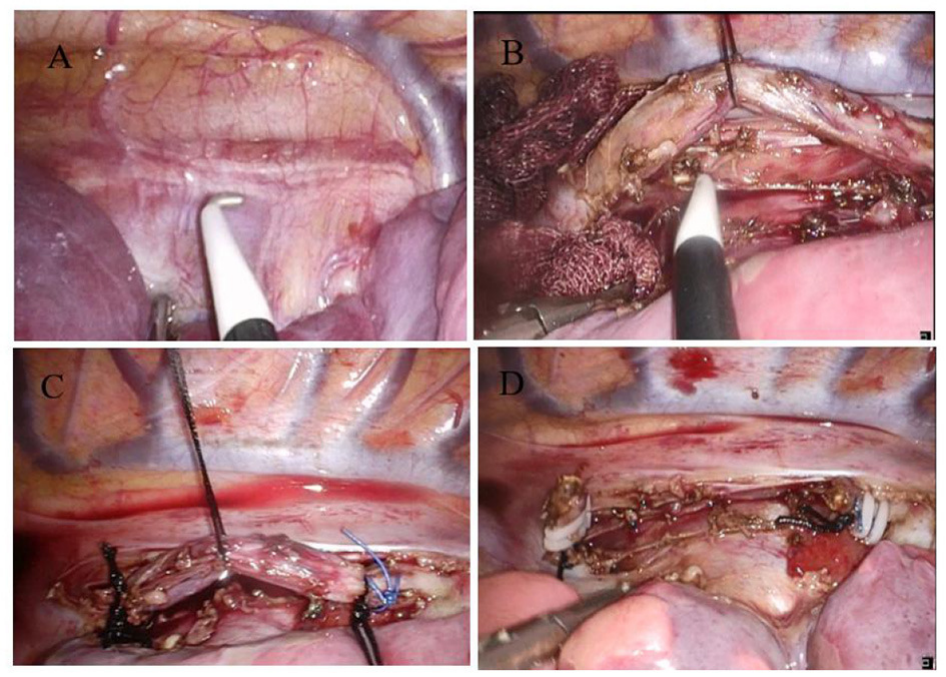
Video-assisted thoracoscopic surgery to cut and suture the fistula. **(A)** CBBF was detected. **(B)** Free CBBF. **(C)** Ligation of CBBF. **(D)** CBBF was cut and sutured.

We discussed the diagnosis and treatment of CBBF with the guardian of the child and signed the informed consent for the operation. The CBBF was cut off and sutured by video-assisted minimally invasive thorascopic surgery. Two endobronchial blockers were used. For one-lung ventilation, one endobronchial blocker was inserted into the right main bronchus under bronchoscope guidance before intubation. After that, a standard 5.5-mm ETT without cuff was inserted into the trachea and connected to the multiport airway adapter. Under bronchoscope guidance, the second endobronchial blocker was inserted into the CBBF through the multiport airway adapter and ETT. The right posterior axillary line in the sixth intercostal space was used as the observation hole, and a 30° 5-mm thoracoscope was inserted. The fourth intercostal space in the right anterior axillary line and the seventh intercostal space in the middle axillary line were the operation holes. A 6-mm-diameter fistula was found at the beginning of the right main bronchus below the tracheal carina (Figure 4A). The fistula runs down the right side of the spine and passes through the diaphragm. By shaking the endobronchial blocker in the fistula, the BBF shaking can be seen under the thoracoscope. The fistula was fully exposed and the whole course of the fistula was free intrathoracic (Figure 4B). After double ligation with No. 10 mousse thread at both ends (Figure 4C), the fistula was double clamped with a hemolock clamp, and the fistula was removed with an ultrasonic knife (Figure 4D). Lastly, the thoracic drainage tube was placed through the seventh intercostal operation hole. The operation lasted 105 min. Tracheal intubation was removed 1 h after the operation. The excised fistula was sent for pathological examination. Microscopically, the specimen presented as a tubular structure of cartilage and muscle, lined with squamous and pseudostratified ciliated columnar epithelial mucosa and submucosal glands. It was considered as tracheal malformation. The lung shadow disappeared, which was demonstrated by chest CT after 6 months of the operation.

## Discussion

CBBF is associated with abnormal embryonic development. Its symptoms can occur from newborn to adult (3, 4). The severity of CBBF is different, which is related to the diameter of the fistula and whether combined with bile tract malformation. The earlier the symptoms appear, the more severe the condition is. This patient began to cough repeatedly at 2 years old but did not expectorate typical yellow-greenish sputum. Typical yellow-greenish sputum is rarely seen in infants. Infants also cannot describe whether the sputum is bitter or not. Therefore, it is easy to miss a diagnosis in infancy. If infants show recurrent pneumonia, yellow-greenish sputum can be seen during sputum suction, and cone-shaped fissure-like protrusion can be seen on chest 3D-CT reconstruction. In this situation, CBBF should be suspected, and relevant examinations should be further improved to make a definite diagnosis.

There are various diagnostic methods for CBBF. The increase of total bile acids in sputum or intratracheal lavage fluid is helpful for the diagnosis of CBBF. Chest CT scan and 3D-CT reconstruction can show the abnormal branches of the trachea. It is difficult to directly show the whole shape of the fistula, but it can evaluate the severity of pulmonary inflammation and guide whether the lobectomy should be performed at the same time. Abdominal CT examination can diagnose primary diseases such as liver abscess and bile duct stones. In addition, some patients can see a gas shadow in the bile duct. Abnormal branches of the trachea and gas shadow in the bile duct are important indirect signs of BBF. In this case, the abnormal branch of the trachea was found in the previous chest CT examination. It was ignored and diagnosis was missed. Percutaneous transhepatic cholangiography (PTC) and endoscopic retrograde cholangiopancreatography (ERCP) can show the origin and course of the fistula. In addition, it can also confirm whether there are other bile duct malformations. At present, PTC and ERCP are the main methods for the diagnosis of acquired bronchobiliary fistula (ABBF) in adults (5). Because the PTC and ERCP are complex and traumatic, it is not widely carried out in children (6, 7). Contrast-enhanced magnetic resonance cholangiography can show the anatomy of the biliary tract and provide physiological or pathological bile flow function information. Compared with conventional magnetic resonance cholangiopancreatography (MRCP), contrast-enhanced magnetic resonance cholangiopancreatography has more obvious diagnostic advantages. However, it needs longer operation time and higher technical requirements (8). Bronchoscopy is the first choice for the diagnosis of CBBF. Through the bronchoscope, we can see the yellow-green liquid overflowing from the fistula, and it will still overflow again after lavage. The bile acid test of lavage fluid is positive, which has an important diagnostic value. The bronchoscopy cannot show the whole course of the fistula. Bronchography can show the course of CBBF. Traditional bronchography involves the catheter being inserted into the trachea through the biopsy hole of the bronchoscope and a contrast agent is injected (9, 10). Li et al. (9) retrospectively analyzed 44 cases of CBBF. It showed that the orifice of CBBF was near the tracheal carina, with 45.4% opening in the right main bronchus, 43.2% opening in the tracheal carina, and 11.4% opening in the left main bronchus. Because the position of the fistula is very high, the contrast medium fistulography is easy to flow to the lower bronchi and alveoli. If the fistula is thin, the fistula is often not displayed, let alone the bile duct. We used the endobronchial blocker to block the orifice of CBBF. The whole shape and course of the BBF could be displayed by injecting contrast agents under x-ray fluoroscopy. There are three advantages to increase the injection pressure of the contrast agents and prolong fluoroscopy time. First of all, in addition to clearly showing the fistula, the left intrahepatic bile duct, the right intrahepatic bile duct, the common hepatic duct, the common bile duct, the gallbladder, and the duodenum can also be displayed in sequence. To determine whether there is a biliary malformation, ERCP cholangiography is not necessary. It can provide an important reference for the choice of surgical methods. Secondly, CBBF can be located by anteroposterior and lateral fluoroscopy. It can help to select the position of the observation hole and the operation hole for the thoracoscopic surgery. Finally, the CBBF tracheal fistula was occluded for angiography, which can avoid contrast media reflux to the bronchus and alveoli.

The main treatment method of CBBF is operation, which depends on the location of the fistula and whether it is combined with other liver and biliary diseases. It includes fistulectomy and plugging under bronchoscopic guidance. In case of CBBF with bile duct malformation, absence of common bile duct, abnormal bile intestinal drainage, and so on, fistula jejunum Roux-en-Y anastomosis, hilar jejunum anastomosis, and gallbladder jejunum anastomosis, among others, are feasible to fully drain bile and avoid fistula recurrence (11–13). The plugging of the fistula under bronchoscope guidance has the advantages of less trauma and quick recovery. At present, it is mainly used in adult ABBF, and there are few reports in children (14–19). Video-assisted thoracoscopic surgery in the treatment of CBBF can provide an unparalleled surgical vision compared to thoracotomy. In addition, it has less trauma and faster postoperative recovery. However, it is rarely reported in children (20–22). The CBBF-ectomy of this case is through mini-incision by video-assisted thoracoscopy surgery. One-lung ventilation was performed by an endobronchial blocker. The BBF was cut off and sutured. At present, the commonly used single lung ventilation technology includes double-lumen bronchial catheter ventilation and endobronchial blocker blocking one side of the main bronchus (23, 24). The smallest double-lumen bronchus tube is 26F. For children under 8 years old, one-lung ventilation with a double-lumen bronchus tube is impossible. The peripheral diameter of an endobronchial blocker is significantly smaller than a double-lumen endobronchial tube. The applicable age range of the endobronchial blocker is wider than the double-lumen endobronchial tube (2, 25, 26). The bronchial orifice of the CBBF was occluded by an endobronchial blocker. It can prevent bile acid from reflux into the trachea when squeezing the fistula during operation. Then, it can avoid asphyxia and chemical inflammatory injury of the lung. The course of the BBF should be confirmed before resection. We can confirm the target fistula along the abnormal branch of the trachea. Besides, the fistula can be seen by shaking the endobronchial blocker.

In conclusion, if the patient has recurrent cough and refractory pneumonia, we should be alert to CBBF. The airway reconstruction can show abnormal tracheal branches and abdominal CT can find a gas shadow in the liver. Bronchography can make a definite diagnosis of CBBF with the help of the bronchoscope and endobronchial blocker. The application of a double endobronchial blocker during operation can not only effectively implement one-lung ventilation for children but also help to determine the course of BBF and prevent bile acid reflux to the lung. It is a simple, feasible, accurate, and reliable diagnostic and treatment method to block the fistula with an endobronchial blocker. Video-assisted thoracoscopic surgery is a safe (with minimal trauma) and effective way to cut off the fistula.

## Data Availability Statement

The original contributions presented in the study are included in the article/supplementary material, further inquiries can be directed to the corresponding author/s.

## Ethics Statement

The studies involving human participants were reviewed and approved by Qingdao Women and Children's Hospital (QFFLL-YJ-2021-07). Written informed consent to participate in this study was provided by the participants' legal guardian/next of kin.

## Author Contributions

ZB was responsible for data interpretation, drafting of the manuscript, and approval of the final version to be published. RC, PX, YR, and KH were responsible for the study concept, data collection and interpretation, revision of the manuscript, and approval of the final version to be published. All authors read and approved the final manuscript.

## Conflict of Interest

The authors declare that the research was conducted in the absence of any commercial or financial relationships that could be construed as a potential conflict of interest.

## Publisher's Note

All claims expressed in this article are solely those of the authors and do not necessarily represent those of their affiliated organizations, or those of the publisher, the editors and the reviewers. Any product that may be evaluated in this article, or claim that may be made by its manufacturer, is not guaranteed or endorsed by the publisher.
